# Is It a Just World After All? Age Differences in the Belief in a Just World Across 57 Societies and the Influences of Cultural Values

**DOI:** 10.1002/ijop.70141

**Published:** 2025-12-07

**Authors:** Tianyuan Li, Niting Guo, Wenhao Luo

**Affiliations:** ^1^ School of Humanities and Social Science Chinese University of Hong Kong Shenzhen China

**Keywords:** collectivism, flexibility, individualism, justice belief, monumentalism

## Abstract

Individuals' belief in a just world is related to a complexity of personal and social consequences. Understanding the development of justice belief can provide valuable information about its determinants and functions across the lifespan. The current study aims to investigate age‐related differences in belief in a just world across adulthood and how the age differences are moderated by cultural values. Responses from 81,543 individuals across 57 societies were analysed. Individual‐level information was obtained from the sixth wave of the World Values Survey, and society‐level individualism and flexibility scores were obtained from Minkov and Kaasa's work on the two‐dimensional model of cultural values. Hierarchical linear modelling was conducted to analyse the data and revealed a robust positive association between age and the belief in a just world. Moreover, society‐level flexibility, but not individualism, strengthened the positive association between age and the belief in a just world. A positive main effect of flexibility on the belief in a just world was also found. The study revealed age‐related differences in justice belief across societies. The findings suggest that the development of the belief in a just world is jointly influenced by individuals' personal motivational needs for justice and the cultural context.

## 1 Is It a Just World After All? Age Differences in the Belief in a Just World Across 57 Societies and the Influences of Cultural Values

Individuals' dispositional belief in a just world reflects their general belief about whether the world is a just place (Dalbert and Donat 2015). A strong belief in a just world can provide a sense of order and security and indicates basic trust in the world. Such belief is also related to multiple personal and social consequences across different age groups (Bartholomaeus and Strelan 2019; Hafer and Sutton 2016). However, in spite of intensive research on the belief in a just world and its related consequences, the factors that contribute to individual differences in justice belief are underexplored (Bartholomaeus and Strelan 2019; Hafer and Sutton 2016). A comprehensive understanding of the age‐related differences in justice belief can provide valuable information about its determinants and functions across the life span, yet very few studies have examined age differences in belief in a just world across adulthood in large and representative samples. To fill this gap, the current study examined age differences in the belief in a just world across adulthood in 57 societies. We also investigated how the age differences were moderated by society‐level cultural values.

## 2 Belief in a Just World and the Expected Age Differences

According to the justice motive theory (Lerner 1980), people are motivated to believe that the world is a just place where everyone gets what he/she deserves. The belief in a just world is a core element of one's worldview and has pervasive impacts on people's daily lives. Extensive research has explored the consequences related to dispositional justice belief and found that the justice belief is related to a mixture of positive and negative personal and social outcomes (for reviews, see Hafer and Sutton 2016; Silva et al. 2024). The initial studies on belief in a just world argue that individuals use the justice belief as a buffer to protect them from threats and uncertainty in an unjust world. The justice belief is a delusion in an unjust world and can lead to irrational and harmful social judgments. People with stronger justice belief are found to be more likely to derogate and devalue innocent victims and support an unfair system to reassure their justice belief (Twardawski et al. 2025). However, further studies indicate that the belief in a just world is not only driven by irrational thinking. Instead, it can function as a valuable personal resource which supports people to face life's adversities in a more optimistic manner (Dalbert and Donat 2015). Stronger justice belief is related to a higher sense of control, better psychological well‐being, and more prosocial behaviours (e.g., Goodwin and Williams 2023; Guo et al. 2022). The pervasive and intricate findings on the correlates of justice belief highlight the importance and complexity of this concept. Understanding the age‐related changes in people's belief in a just world and how the age differences are shaped by the sociocultural environment could shed critical light on the developmental foundation of justice belief and provide new insights to comprehend the complex nature of justice belief.

Existing studies have examined the influence of the belief in a just world for participants at different life stages from adolescence to late adulthood (e.g., Oppenheimer 2006; Zhang and Zhang 2015), but the age‐related differences in the justice belief remain underexplored (Bartholomaeus and Strelan 2019). Studies that have investigated the development of the justice belief predominantly focused on the period from adolescence to early adulthood (e.g., Oppenheimer 2006; Sun et al. 2024; Thomas et al. 2024) and revealed mixed results. Research on the life‐span development of justice belief across adulthood is still lacking.

A commonly believed developmental origin of the justice belief is a *need* to believe that the world is a just place (Hafer and Sutton 2016). According to the justice motive theory (Lerner 1980), the justice belief enables individuals to face the world as though it were orderly and stable, and provides people with a heightened sense of certainty, meaningfulness, and personal control (Goodwin and Williams 2023; Yu et al. 2018). Based on previous research on lifespan development, such a need may become more salient while people age. According to the life‐span theory of control (Schulz and Heckhausen 1996), with increasing age and the inevitable decline of physical functioning, individuals' ability to cope with life challenges using primary control strategies (i.e., directing attempts to change the external environment to fit one's needs) becomes limited. Therefore, they are more likely to employ secondary control strategies (i.e., adjusting one's own perceptions to fit external conditions) and adjust their perception of the world to restore the thwarted sense of control. Belief in a just world could lead people to view the outcomes in their lives as consequences of their own actions (Dalbert and Donat 2015) and has been consistently found to contribute to higher perceived control (e.g., Goodwin and Williams 2023; Yu et al. 2018). Thus, believing in a just world can be a great secondary control strategy to maintain the sense of control, particularly with the advancement of age.

In addition, the socioemotional selectivity theory suggests that as people age and perceive time as more limited, they place greater emphasis on emotionally meaningful goals and meaningful social connections (Carstensen 2021). The belief in a just world denotes basic trust in other people and the social system (Dalbert and Donat 2015; Schmitt et al. 2023), providing reassurance that the social relationships and engagement with society are safe and trustworthy. Through an enhanced level of identification and connectedness with society, the belief in a just world can thereby help individuals attain emotional meaningfulness in their lives, which becomes increasingly important while they age.

Meanwhile, as suggested by the terror management theory (Greenberg et al. 1986), uplifted identification with one's social groups or cultural values could help ease the anxiety brought by mortality, as one's social groups and values could surpass individual lives. Previous research has consistently found that, although older adults are chronologically closer to mortality, they reported lower levels of death anxiety compared to middle‐aged adults (Gesser et al. 1988). This paradox suggests that older adults are very effective and successful in managing mortality threats. An enhanced level of belief in a just world can be one of the strategies that older adults use to cope with death anxiety. The belief that the world is a just place could strengthen older adults' social identity and the endorsement of social values (Silva et al. 2024), thus contributing to the sense of symbolic immortality and ameliorating the mortality threat.

To summarise, different theoretical accounts jointly suggest that older adults would be more motivated to hold the belief in a just world. Such a belief could work as a secondary coping strategy and be particularly helpful for individuals to obtain emotional meaningfulness and social connectedness with the advancement of age. Thus, our first hypothesis is:

Hypothesis 1 *Older age is related to* a *stronger belief in a just world*.

It is important to note that the current research defines the belief in a just world in terms of an overarching evaluation about the fairness of other people and the external world without differentiating the recipient of the justice. This definition aligns with the *global* belief in a just world concept proposed by Lipkus (1990), which refers to a generalised belief that the world operates on principles of fairness. We are aware that many recent studies distinguish between *personal* belief in a just world (i.e., perceived justice to oneself) and *general* belief in a just world (i.e., perceived justice to others) (Dalbert 1999; Dalbert and Donat 2015). Compared to the general belief in a just world, the personal belief in a just world is found to be more critical for personal well‐being (Qiu and Tang 2025). However, studies have also found that the two types of justice belief correlated highly with each other and the general belief in a just world is largely dependent on one's personal experiences as well (Kong et al. 2021). As illustrated above, the expected age‐related changes in the belief in a just world are relevant to the overall evaluation of the external world, but not a specific recipient (i.e., self vs. others) of justice, thus we adopt the global definition and do not make a deliberate distinction between personal and general belief in a just world in the current study.

Moreover, the current research aims to investigate age differences in the belief in a just world across cultures. Previous research has identified consistent cultural differences when personal and general beliefs in a just world are differentiated. Individuals tend to endorse the general justice belief more than the personal one in collectivistic societies (Sun et al. 2024; Wu et al. 2011), and endorse the personal justice belief more than the general one in individualistic cultures (e.g., Sutton and Douglas 2005). Adopting the global definition of belief in a just world could minimise the cultural variations and provide a more meaningful basis for cross‐cultural comparison.

## 3 The Influence of Culture Values

Besides the motivational need to believe in a just world, individuals' justice belief is also influenced by social learning and justice‐related experiences in real life (Hafer and Sutton 2016; Thomas 2022). In particular, Thomas (2022) proposed the justice capital framework and argued that justice belief was the product of individuals' everyday justice experiences based on one's justice capital. Bartholomaeus et al. (2023) provided cross‐cultural evidence showing that access to justice influences justice belief. Bartholomaeus (2025) further linked distinct justice belief profiles to demographic and justice‐related experiences in a large sample. Longitudinal studies with adolescents also showed that young people's justice belief systematically changed with everyday encounters with fairness and unfairness (e.g., Thomas et al. 2024). Cultural values serve as socially approved guidelines that shape how people treat and interact with others (Hofstede 2011). Society‐level cultural values can have a systematic influence on daily social interactions and justice‐related experiences within the society, which could further influence individuals' belief in a just world, as well as age differences in justice belief.

Extensive research has been conducted to identify the key dimensions of cultural values across societies. Among different models, Hofstede's framework has been very influential (Hofstede 2011). The model proposes six dimensions of cultural values, namely individualism–collectivism, power distance, masculinity–femininity, uncertainty avoidance, long‐term orientation, and indulgence. However, some recent studies could not replicate the six‐dimensional model, and some dimensions are criticised for limited predictive accuracy (Minkov 2017). Building on Hofstede's framework and data from the World Values Survey (Inglehart et al. 2014), Minkov and Kaasa (2022) further proposed a two‐dimensional model. The two‐dimensional model has gained growing consensus in the field and can be applied to over 100 countries (e.g., Fog 2021). The new model identifies individualism–collectivism and flexibility–monumentalism as two fundamental dimensions of cultural values. The individualism dimension reflects the extent to which personal autonomy versus group cohesion and conformity to societal norms are valued in the society. The flexibility dimension, rooted in the long‐term orientation dimension proposed by Hofstede, characterises how different cultures approach change and adaptation. In flexible societies, people tend to expect and prepare for changes in life, embrace pragmatic solutions, promote delayed gratification, and maintain adaptability in the face of challenges. Conversely, monumental societies emphasise maintaining consistent self‐identity and immutable values. In the current research, we adopt the two‐dimensional view of cultural values and expect both individualism and flexibility to influence the belief in a just world in a society and the related age differences.

First, society‐level individualism can be negatively related to the belief in a just world in society. People in collectivistic cultures prioritise group harmony, social cohesion, and belongingness to social groups (Schermer et al. 2023), so they may be more accepting of some seemingly unfair treatments to individuals for the sake of larger collectivistic welfare. Moreover, as a result of the awareness of interconnectedness between oneself and others in society, the perception of social justice tends to function in a more positive way in collectivistic cultures. For instance, Wu et al. (2011) showed that individuals from collectivistic cultures reported a high general belief in a just world, which further contributed to individuals' psychological resilience. In contrast, people in individualistic cultures emphasise personal autonomy, achievements, and competition with others (Schermer et al. 2023), so they are more likely to attribute social injustice when their personal benefits are thwarted. While several previous studies have reported the negative association between individualistic values and the belief in a just world based on data at the individual level (e.g., Oppenheimer 2006), the current study can test this relationship at the societal level. Thus, our second hypothesis is:

Hypothesis 2 *Society‐level individualism is negatively associated with the belief in a just world*.

More importantly, society‐level individualism can also influence age differences in the belief in a just world. As personal autonomy and self‐reliance are valued in individualistic cultures (Minkov and Kaasa 2022), maintaining personal agency is crucial for individuals in such cultures throughout their lifespan. When older adults face age‐related declines in primary control capabilities, those in individualistic cultures may experience a greater psychological threat to their sense of autonomy compared to their counterparts in collectivistic cultures. Consequently, they may be more motivated to employ justice belief as an important secondary control strategy to maintain their sense of personal control. Thus, we hypothesize that:

Hypothesis 3 *The positive association between age and justice belief would be more apparent in societies with higher individualism*.

Meanwhile, we expect society‐level flexibility to be positively related to the belief in a just world in the society. First, individuals in flexible cultures are more accepting of changes in life and are readier to adapt themselves to suit the environment (Zhou et al. 2024), so they are more likely to attribute difficulties and challenges in life to internal reasons (e.g., I need to change) rather than the environment (e.g., the social system is unjust). Second, long‐term orientation and delayed gratification are prioritised in flexible cultures (Minkov and Kaasa 2022). Some life experiences may seem unfair in the short term but can be paid off in the long run. Individuals in flexible cultures may tend to review their life experiences in a more extended time frame and, thus, are more likely to report a higher level of justice belief. Previous research also supported the positive association between delayed gratification and the belief in a just world (Lerner 1980), as well as that between long‐term orientation and the perception of justice (Ha and Lee 2024). Thus, our fourth hypothesis is:

Hypothesis 4 *Society‐level flexibility is positively associated with the belief in a just world*.

Moreover, society‐level flexibility is expected to strengthen the positive association between older age and higher levels of belief in a just world. Flexibility values encourage individuals to adapt and change oneself in response to life challenges (Minkov and Kaasa 2022). The cultural emphasis on adaptability may further promote older adults' psychological adjustments to age‐related changes in flexible cultures. Thus, older adults in flexible cultures are more likely to develop a stronger justice belief as an adaptive coping mechanism to restore their sense of control and meaningfulness. In contrast, individuals in monumental cultures are encouraged to maintain a consistent identity and enduring values and beliefs across different situations (Minkov and Kaasa 2022), so age differences in justice beliefs can be less apparent in monumental cultures. Hence, our fifth hypothesis is:

Hypothesis 5 *The positive association between age and justice belief would be more apparent in societies with higher flexibility*.

## 4 The Current Study

To conclude, the current study aimed to examine age differences in the belief in a just world across 57 societies. Moreover, we also examined how the belief in a just world and the related age differences were influenced by society‐level individualism and flexibility. As individuals' demographic background and the developing status and income inequality of society can also influence one's belief in a just world (Vargas‐Salfate et al. 2018; Wang et al. 2024), these variables were controlled in all the analyses.

## 5 Method

### 5.1 Sample

The current study used the data from the sixth wave of the World Values Survey (WVS6; Inglehart et al. 2014), which was collected from 2010 to 2014. A total of 81,543 participants (Mean_age_ = 41.72, SD_age_ = 16.41, ranging from 16 to 102 years; 52% females) from 57 societies with complete data for age, sex, health, household income, and the belief in a just world measure were included in the analyses. The sample characteristics in each society were presented in Table S1. As the study used publicly available secondary data, we did not apply for additional ethical approval.

### 5.2 Measures

#### 5.2.1 Individual‐Level Variables

##### 5.2.1.1 Belief in a Just World

Belief in a just world was measured by a single item in WVS6. Each participant responded to the question ‘Do you think most people would try to take advantage of you if they got a chance, or would they try to be fair?’ from 1 (*people would try to take advantage of you*) to 10 (*people would try to be fair*). The item is adopted from Smith's (1997) misanthropy scale, assessing the generalised perception of other people's fairness. Consistent with the definition of the global belief in a just world, the item did not specify the target of the justice evaluation. The pronoun ‘*you*’ could refer to either oneself or other people in general. Moreover, the belief in a just world mainly reflects individuals' uncertainty about interactions with other people (Zhang and Zhang 2025). Thus, assessing the perception about whether most people are fair captures the core of the global belief in a just world. Similar items about fairness perception are also found in both the personal belief in a just world scale (i.e., ‘I am usually treated fairly’) and the general belief in a just world scale (i.e., ‘I think people try to be fair when making important decisions’) (Dalbert 1999).

##### 5.2.1.2 Demographic Information

Participants' age, sex (−1 = *male*, 1 = *female*), health, and household income were collected in WVS6. The sample covered a wide age range, and the two sexes were approximately balanced. Participants rated their health on a 4‐point scale from 1 (*very good*) to 4 (*poor*). The score was reversed so that a higher score indicated better health. Considering the vast differences in economic development and currency systems across the societies, household income was measured in a relative manner. Participants rated their own household income in comparison to all the other households in their society on a 10‐point scale from the lowest income decile (1) to the highest income decile (10).

#### 5.2.2 Society‐Level Variables

##### 5.2.2.1 Individualism and Flexibility

Individualism and flexibility scores for each society were obtained from Minkov and Kaasa's (2022) study, which provided individualism–collectivism and flexibility–monumentalism indices for 102 societies based on data from the WVS. The individualism score reflects the attitudes toward personal liberty and individual rights, capturing the extent to which societies prioritise personal freedom and individual autonomy over collective norms. The flexibility score reflects the emphasis on self‐sufficiency, delay of gratification, and adaptability in contrast to consistency, tradition, and fixed values. For the 57 societies covered in the current study, the individualism score ranges from −291 to 182, and the flexibility score ranges from −285 to 234. Higher scores indicate higher levels of individualism or flexibility values in corresponding societies.

##### 5.2.2.2 Developing Status

The Human Development Index (HDI) of each society in 2012 (United Nations Development Programme [UNDP] 2013) was adopted to reflect each society's developing status. The HDI is a summary measure calculated based on life expectancy, average education attainment, and gross national income per capita of a society. The index ranges from 0 to 1, with a larger number indicating a higher level of development.

##### 5.2.2.3 Income Inequality

The income Gini coefficient of each society from 2010 to 2021 was mainly adopted from the UNDP (2022). For eight societies not covered in the UNDP report, we obtained their Gini coefficient from the Standardised World Income Inequality Database (Solt 2020; see Table S1 for details). The Gini coefficient ranges from 0 to 100, with 0 indicating perfect equality and 100 indicating absolute inequality.

### 5.3 Data Analysis

The data were analysed by hierarchical linear modelling (HLM) using the software HLM (Raudenbush and Bryk 2002). Individual‐level data were considered as level‐1 variables, and society‐level data were considered as level‐2 variables. Before entering the variables into the models, we standardised age across the entire sample, while individual health and household income were standardised within each society. All society‐level variables were standardised across societies. To start with, we estimated an unconditional model without any predictors to examine the within‐ and between‐society variations of the belief in a just world. Four HLM models were then constructed to test the hypotheses. In all models, age was entered as a level‐1 predictor of the belief in a just world. Sex, health, and household income were included as level‐1 covariates, while HDI and Gini coefficient were added as level‐2 covariates. Model 1 did not contain either individualism or flexibility in the model. Models 2 and 3 tested the effect of individualism and flexibility, respectively, and Model 4 tested the effect of individualism and flexibility simultaneously. The detailed equations of the HLM models were presented in Supporting Information. Simple slope analyses were conducted to further illustrate the significant moderating effects of the cultural values.

## 6 Results

Table S1 summarised the descriptive information across 57 societies. The results of the HLM models were presented in Table 1. The results of the unconditional model showed that the grand mean of justice belief was 5.677 across all societies. There was significant between‐society variance in justice belief (τ_00_ = 0.674, *p* < 0.001), indicating that justice belief varies substantially across societies.

**TABLE 1:** Four HLM models testing the age differences in the belief in a just world with or without society‐level cultural values.

Parameters	Unconditional model	Model 1	Model 2	Model 3	Model 4
Regression coefficients					
Intercept (γ_00_)	5.677***	5.651***	5.650***	5.650***	5.650***
Age (γ_10_)	—	0.075**	0.074**	0.074**	0.074**
Sex (γ_20_)	—	0.075***	0.075***	0.075***	0.075***
Health (γ_30_)	—	0.170***	0.171***	0.171***	0.171***
Income (γ_40_)	—	0.241***	0.241***	0.241***	0.241***
HDI (γ_01_)	—	0.195	0.376	0.034	0.230
Gini (γ_02_)	—	−0.004	−0.011	0.074	0.075
Individualism (γ_03_)	—	—	−0.257	—	−0.305
Flexibility (γ_04_)	—	—	—	0.294*	0.330*
Interaction: age × HDI (γ_11_)	—	0.027	−0.003	−0.003	−0.026
Interaction: age × Gini (γ_12_)	—	−0.026	−0.025	−0.012	−0.012
Interaction: Age × individualism (γ_13_)	—	—	0.042^+^	—	0.035
Interaction: age × flexibility (γ_14_)	—	—	—	0.055*	0.051*
Variance components					
Residual^a^ (*σ* ^2^)	6.369	6.193	6.193	6.193	6.193
Intercept (τ_00_)	0.674***	0.638***	0.624***	0.589***	0.562***
Slope of age (τ_11_)	—	0.019***	0.017***	0.017***	0.016***
Model summary					
Deviance statistic	382,671.782	380,723.502	380,725.552	380,720.987	380,722.855
Number of estimated parameters	2	16	16	16	16

Abbreviations: Gini, income Gini coefficient; HDI, human development index.

^a^ The significance of the residual component in each model was not tested.

*p < 0.05, **p < 0.01, ***p < 0.001, ^+^ *p* = 0.058.

Supporting Hypothesis 1, Model 1 revealed a significant positive association between age and justice belief (γ_10_ = 0.075, *p* = 0.001), indicating older adults reported higher levels of belief in a just world. The positive age effect remained significant in Models 2 to 4 when the effects of cultural values were considered.

**FIGURE 1:**
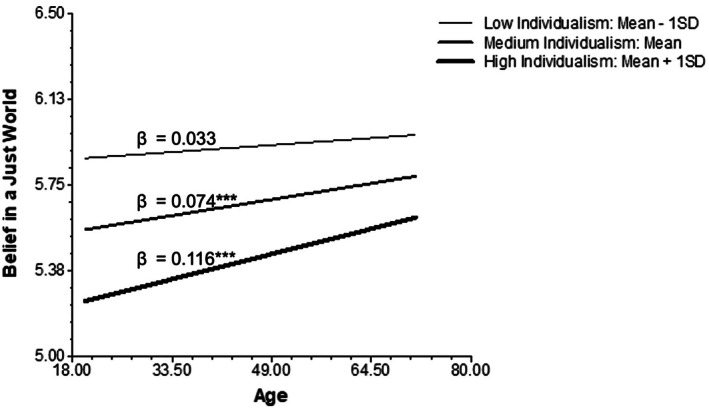
Age differences in the belief in a just world moderated by society‐level individualism.

Model 2 examined both the main effect of society‐level individualism on the belief in a just world and the moderating effect of individualism on the age‐justice belief association. The main effect of individualism on belief in a just world was not significant (γ_03_ = −0.257, *p* = 0.168), so Hypothesis 2 was not supported. Meanwhile, consistent with Hypothesis 3, a significant positive interaction between individualism and age was observed (γ_13_ = 0.042, *p* = 0.058), but the effect was only marginally significant. As expected, society‐level individualism tended to strengthen the positive association between age and the belief in a just world. We still conducted simple slope analyses to further illustrate the moderating effect of individualism (see Figure 1). We observe a significant positive association between age and the belief in a just world in societies with average individualism values (γ_10_ = 0.074, *p* < 0.001). Consistent with Hypothesis 3, the positive association became stronger in societies with high individualism (mean + 1SD; γ_10_ = 0.116, *p* < 0.001) but was attenuated and became nonsignificant in societies with low individualism (mean—1SD; γ_10_ = 0.033, *p* = 0.269).

**FIGURE 2:**
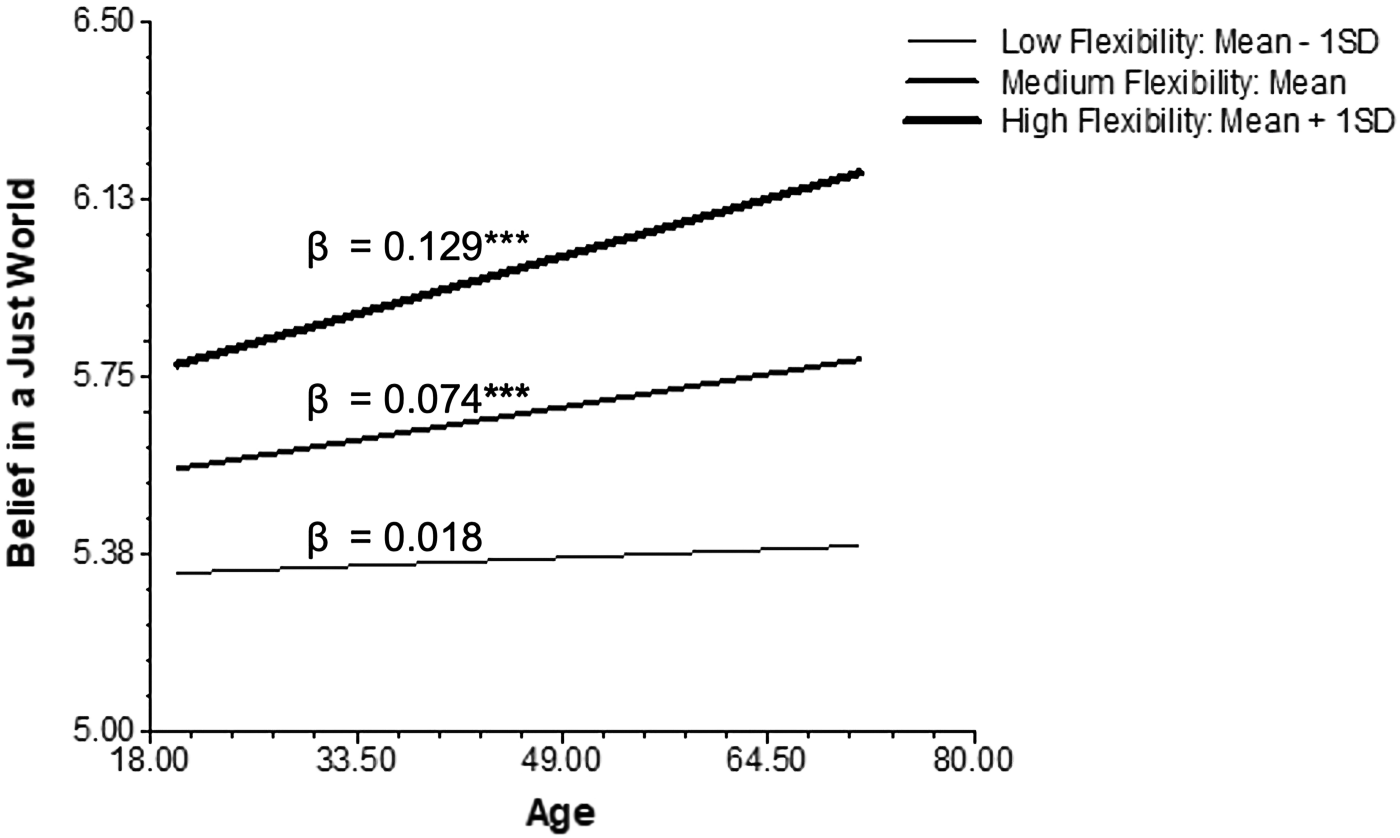
Age differences in the belief in a just world moderated by society‐level flexibility.

Similarly, Model 3 examined both the main effect of society‐level flexibility on the belief in a just world and the moderating effect of flexibility on the age‐justice belief association. There was a significant positive main effect of flexibility on belief in a just world (γ_03_ = 0.294, *p* = 0.048), supporting Hypothesis 4 that higher society‐level flexibility was related to higher justice belief in the society. Moreover, supporting Hypothesis 5, a positive interaction between flexibility and age was identified (γ_13_ = 0.055, *p* = 0.016), suggesting that society‐level flexibility strengthened the positive association between age and the belief in a just world. Simple slope analyses were conducted to further examine the moderating effect of flexibility (see Figure 2). There was a significant positive association between age and the belief in a just world in societies with average flexibility (γ_10_ = 0.074, *p* < 0.001). The positive association intensified in societies with high flexibility (mean + 1SD; γ_10_ = 0.129, *p* < 0.001) but diminished and became nonsignificant in societies with low flexibility (mean—1SD; γ_10_ = 0.018, *p* = 0.563).

Model 4 examined the effects of individualism and flexibility simultaneously. The main effects of age (γ_10_ = 0.074, *p* = 0.001) and flexibility (γ_04_ = 0.330, *p* = 0.018) on the belief in a just world remained significant, as well as the age × flexibility interaction (γ_14_ = 0.051, *p* = 0.025). However, the main effect of individualism (γ_03_ = −0.305, *p* = 0.093) and the age × individualism interaction on the belief in a just world (γ_13_ = 0.035, *p* = 0.132) were both nonsignificant.

## 7 Discussion

This study took the first step to investigate age differences in the belief in a just world and the moderating effects of cultural values in 57 societies. The significant cross‐societal variability in the belief in a just world underscores the importance of examining justice beliefs in a cross‐cultural framework. The results revealed a robust positive association between age and the belief in a just world across different models. Moreover, society‐level flexibility was also related to a high level of justice belief in the society, and it was found to strengthen the positive association between age and the justice belief. The results also showed a trend for society‐level individualism to strengthen the positive association between age and the justice belief, but the effect was not as robust as that for flexibility. The findings shed new light on the development of justice belief and the influencing factors.

The general pattern of age‐related differences in the belief in a just world across different societies supports our first hypothesis. Older age is associated with higher levels of justice belief. While some previous studies have also reported a positive association between age and the justice belief across adulthood (Harding et al. 2020), they were based on small samples. The current study revealed the relationship between age and the justice belief based on a more generalizable sample across 57 societies. Older adults' higher justice belief reflects the increased motivational need for justice in old age. Facing limited future time and inevitable physical declines in later adulthood, older adults' heightened belief in a just world can help them maintain a sense of control, obtain emotional meaningfulness and social connectedness, and manage death anxiety (e.g., Carstensen 2021; Greenberg et al. 1986; Schulz and Heckhausen 1996). The implications of older adults' higher justice belief can be multifaceted. On the one hand, older adults' stronger justice belief may help explain their higher prosocial tendencies compared to their younger counterparts (e.g., Li and Siu 2021). Moreover, the results support that older adults tend to hold more positive beliefs about other people and society, which aligns with previous findings (e.g., Li and Fung 2013). Such positive beliefs can greatly contribute to social capital and solidarity, highlighting older adults' constructive role in society. On the other hand, the stronger belief in a just world may also lead older adults to support the status quo even when the current system is flawed (Dalbert and Donat 2015). Such a tendency can help explain older adults' relatively high level of conservation (Cornelis et al. 2009), and it may hinder social development in certain circumstances. Future research may further explore the important consequences related to older adults' higher levels of justice belief.

The current findings also support that the belief in a just world is influenced by social learning and perception of actual justice‐related life experiences guided by cultural values. According to Thomas's (2022) justice capital framework, individuals' justice beliefs are shaped by their justice‐related experiences in everyday life, and factors that systematically influence the experiences of justice form one's justice capital. Similarly, Hafer and Sutton's (2016) framework also argues that social learning and life experiences contribute to individuals' justice beliefs in addition to the motivational need. In particular, the current results reveal that the flexibility values in a society not only influence the average level of justice belief in a society (Hypothesis 4) but also strengthen the positive association between age and justice belief (Hypothesis 5). Both effects of flexibility are robust with or without individualism in the same model. In flexible societies, long‐term orientation and the ability to adapt oneself to suit the environment, including the social system, are valued (Minkov and Kaasa 2022). Through cultural socialisation, individuals acquire the tendency to evaluate life experiences using a longer time frame. They also learn to attribute to themselves instead of the unjust social system when encountering life difficulties. Thus, individuals in flexible cultures are more likely to perceive the world as a just place. Previous research has found at the individual level that cognitive flexibility could facilitate adaptive coping and positive reinterpretation of life challenges (Cha et al. 2021). Our findings further corroborate that flexibility values are positively related to justice belief at the societal level.

Meanwhile, the emphasis on adaptability in flexible societies also intensifies the positive association between age and the belief in a just world. In flexible societies, personal values and beliefs are considered malleable depending on situations rather than consistent throughout the life span (Minkov and Kaasa 2022). Thus, older adults in flexible cultures are more likely to change their justice beliefs to cope with the age‐related changes in later adulthood. The results highlight the importance of flexibility values in facilitating older adults to adjust to age‐related changes. A recent study has found that individuals from flexible cultures showed better adjustment when facing realistic challenges and adversity (Zhou et al. 2024). Future studies can further investigate whether flexibility values could moderate other age‐related adaptations and whether flexibility values could further contribute to older adults' physical and psychological well‐being.

An interesting note is that the interaction effect of age and flexibility on the belief in a just world could also be understood from another perspective. Age may serve as a moderator that strengthens the association between individuals' flexibility values and the belief in a just world. While the current study could not directly examine this possibility as we did not assess flexibility values at the individual level, future research could obtain relevant information and investigate the reciprocal moderating effects of age and cultural values on the belief in a just world.

Compared to flexibility, the effects of individualism on the belief in a just world and the age differences are less robust. First, unlike previous studies at the individual level (e.g., Oppenheimer 2006), the current study did not find a significant negative association between individualism and the justice belief at the societal level. Meanwhile, although the results of Model 3 and the subsequent simple slope analyses support Hypothesis 3 and suggest that the positive association between age and belief in a just world was stronger in societies with higher individualism values, the moderation effect of individualism was only marginally significant and became nonsignificant when flexibility was added to the model. The results suggest that, among the two dimensions of cultural values, flexibility is more fundamental in shaping the development of justice belief compared to individualism. The emphasis on long‐term orientation and personal adaptability is more influential to people's perception of justice and the related age differences compared to the focus on autonomy. This contrast of results deepens our understanding of the nature of justice belief and the differential effects of the two cultural dimensions in Minkov and Kaasa's model (2022).

The current study also has limitations. First, based on the cross‐sectional data, we cannot exclude cohort effects from the identified age differences. Future studies can adopt a longitudinal design to further examine the developmental changes in the belief in a just world. Second, the belief in a just world was assessed by a single item in the current study. While single‐item measures are a common choice in large‐scale studies (e.g., Li and Fung 2013) and could exhibit comparable reliability and validity to multi‐item measures (Mund et al. 2023), the current item only assessed individuals' global belief in a just world, and may not reflect the multiple facets of the concept. While the current theoretical framework is more relevant to the global belief in a just world, individuals' justice beliefs can be further differentiated. For example, extensive research has investigated and contrasted personal and general belief in a just world (Dalbert 1999; Qiu and Tang 2025). Other studies have differentiated beliefs in distributive and procedural justice beliefs (Lucas et al. 2011). Future research may use multi‐item scales and examine how age and cultural values jointly influence each facet of the justice belief at a more nuanced level.

To conclude, the current study depicts the relationship between age and the belief in a just world, as well as the moderating effects of cultural values across 57 societies. It contributes to the theoretical understanding of the development of justice belief. The results highlight the importance of the joint influence of the motivational need and cultural context in explaining the age differences in the belief in a just world. Future research is encouraged to further investigate the various factors that contribute to the development of individuals' belief in a just world and the psychological and social impacts of older adults' enhanced levels of justice belief.

## Author Contributions

Tianyuan Li and Niting Guo contributed equally to this work. Tianyuan Li contributed to the conceptualization, methodology, supervision, funding acquisition, writing of the original draft, and revision of the manuscript. Niting Guo was responsible for data analysis, methodology, writing of the original draft, and revision of the manuscript. Wenhao Luo contributed to data analysis and revision of the manuscript.

## Funding

This work was supported by the National Natural Science Foundation of China (72504239) and the Basic and Applied Basic Research Foundation of Guangdong Province (2023A1515110295).

## Ethics Statement

The authors have nothing to report.

## Conflicts of Interest

The authors declare no conflicts of interest.

## Supporting information


**Data S1:** Supporting Information.

## Data Availability

All data related to the current study were accessible at the Open Science Framework (https://osf.io/vkt36/?view_only=6f000106ecc3403d8c8035397a777d89). The questionnaire and original data of the World Values Survey are available from its official website (https://www.worldvaluessurvey.org/wvs.jsp).
