# Supplementary material for: Is It a Just World After All? Age Differences in the Belief in a Just World Across 57 Societies and the Influences of Cultural Values
Source: Int J Psychol. 2025 Dec 7;61(1):e70141. doi: 10.1002/ijop.70141 (PMC12683083; doi:10.1002/ijop.70141)
Supplement: Supplementary file 1 — Data S1: Supporting Information. [file IJOP-61-e70141-s001.docx]

**Supplementary Materials**

**HLM equations for Model 1**

Level 1 equation:

Justice belief_ij_ = β_0j_ + β_1__j_(*Age_ij_* - $\bar{Age}$) + β_2j_*Sex_ij_* + β_3j_(*Health_ij_* - $\overline{{Health}_{j}}$)+ β_4j_(*Income_ij_* - $\overline{{Income}_{j}}$)+ *r*_ij_,

Level 2 equations:

β_0j_ = γ_00_ + γ_01_ (*HDI_j_* - $\bar{HDI}$)+ γ_02_(*Gini_j_* - $\overline{Gini}$)+ *u*_0j_,

β_1j_ = γ_10_ + γ_11_(*HDI_j_* - $\bar{HDI}$) + γ_12_(*Gini_j_* - $\overline{Gini}$) + *u*_1j_,

β_2j_ = γ_20_ + *u*_2j_,

β_3j_ = γ_30_ + *u*_3j_,

β_4j_ = γ_40_ + *u*_4j_.

**HLM equations for Model 2**

Level 1 equation:

Justice belief_ij_ = β_0j_ + β_1j_(*Age_ij_* - $\bar{Age}$) + β_2j_*Sex_ij_* + β_3j_(*Health_ij_* - $\overline{{Health}_{j}}$) + β_4j_(*Income_ij_* - $\overline{{Income}_{j}}$) + *r*_ij_,

Level 2 equations:

β_0j_ = γ_00_ + γ_01_(*HDI_j_* - $\bar{HDI}$) + γ_02_(*Gini_j_* - $\overline{Gini}$) + γ_03_(*IDV_j_* - $\bar{IDV}$)+ *u*_0j_,

β_1j_ = γ_10_ + γ_11_(*HDI_j_* - $\bar{HDI}$) + γ_12_(*Gini_j_* - $\overline{Gini}$) + γ_13_(*IDV_j_* - $\bar{IDV}$) + *u*_1j_,

β_2j_ = γ_20_ + *u*_2j_,

β_3j_ = γ_30_ + *u*_3j_,

β_4j_ = γ_40_ + *u*_4j_.

**HLM equations for Model 3**

Level 1 equation:

Justice belief_ij_ = β_0j_ + β_1j_(*Age_ij_* - $\bar{Age}$) + β_2j_*Sex_ij_* + β_3j_(*Health_ij_* - $\overline{{Health}_{j}}$) + β_4j_(*Income_ij_* - $\overline{{Income}_{j}}$) + *r*_ij_,

Level 2 equations:

β_0j_ = γ_00_ + γ_01_(*HDI_j_* - $\bar{HDI}$) + γ_02_(*Gini_j_* - $\overline{Gini}$) + γ_03_ (*FLX_j_* - $\bar{FLX}$)+ *u*_0j_,

β_1j_ = γ_10_ + γ_11_(*HDI_j_* - $\bar{HDI}$) + γ_12_(*Gini_j_* - $\overline{Gini}$) + γ_13_ (*FLX_j_* - $\bar{FLX}$) + *u*_1j_,

β_2j_ = γ_20_ + *u*_2j_,

β_3j_ = γ_30_ + *u*_3j_,

β_4j_ = γ_40_ + *u*_4j_.

**HLM equations for Model 4**

Level 1 equation:

Justice belief_ij_ = β_0j_ + β_1j_(*Age_ij_* - $\bar{Age}$) + β_2j_*Sex_ij_* + β_3j_(*Health_ij_* - $\overline{{Health}_{j}}$) + β_4j_(*Income_ij_* - $\overline{{Income}_{j}}$) + *r*_ij_,

Level 2 equations:

β_0j_ = γ_00_ + γ_01_(*HDI_j_* - $\bar{HDI}$) + γ_02_(*Gini_j_* - $\overline{Gini}$) + γ_03_(*IDV_j_* - $\bar{IDV}$) + γ_04_(*FLX_j_* - $\bar{FLX}$) + *u*_0j_,

β_1j_ = γ_10_ + γ_11_(*HDI_j_* - $\bar{HDI}$) + γ_12_(*Gini_j_* - $\overline{Gini}$) + γ_13_(*IDV_j_* - $\bar{IDV}$) + γ_14_(*FLX_j_* - $\bar{FLX}$) + *u*_1j_,

β_2j_ = γ_20_ + *u*_2j_,

β_3j_ = γ_30_ + *u*_3j_,

β_4j_ = γ_40_ + *u*_4j_.

(IDV = individualism; FLX = flexibility)

In the models, β_1j_ represent the effect of age on justice belief; γ_03_ and γ_04_ represent the main effects of IDV and/or FLX on justice belief; γ_13_ and γ_14_ represent the moderating effects of IDV and/or FLX on the slope of age on justice belief.

**Table S1**

*Descriptive Statistics Across 57 Societies*

|  |  |  |  |  |  | Age | | |  |  |  |
| --- | --- | --- | --- | --- | --- | --- | --- | --- | --- | --- | --- |
| Society | N | IDV | FLX | HDI | Gini | Mean (SD) | Min | Max | Sex^1^ | Health | Income |
| Algeria | 1,127 | -83 | -95 | 0.71 | 27.6 | 37.48 (14.90) | 18 | 87 | 0.49 | 2.19 (0.88) | 4.72 (2.01) |
| Azerbaijan | 1,002 | -88 | 55 | 0.73 | 29.3^2^ | 41.13 (15.23) | 18 | 85 | 0.50 | 2.26 (0.78) | 5.32 (1.40) |
| Argentina | 959 | 0 | -80 | 0.81 | 42.3 | 43.11 (17.57) | 18 | 92 | 0.53 | 2.04 (0.84) | 5.31 (1.50) |
| Australia | 1,392 | 83 | 41 | 0.94 | 34.3 | 53.25 (16.48) | 18 | 95 | 0.56 | 1.95 (0.80) | 4.90 (2.11) |
| Armenia | 1,042 | -127 | -38 | 0.73 | 25.2 | 46.42 (17.81) | 18 | 85 | 0.66 | 2.71 (0.89) | 4.05 (1.86) |
| Brazil | 1,438 | -56 | -83 | 0.73 | 48.9 | 42.60 (16.24) | 18 | 92 | 0.62 | 2.09 (0.79) | 4.41 (2.07) |
| Belarus | 1,496 | -13 | 27 | 0.79 | 24.4 | 44.31 (17.05) | 18 | 86 | 0.56 | 2.73 (0.74) | 4.75 (1.72) |
| Chile | 892 | -8 | -153 | 0.82 | 44.9 | 44.28 (16.15) | 18 | 85 | 0.50 | 2.13 (0.74) | 4.83 (1.73) |
| China | 1,908 | -31 | 134 | 0.70 | 38.2 | 43.54 (14.82) | 18 | 75 | 0.50 | 2.15 (0.85) | 4.46 (1.85) |
| Colombia | 1,476 | -81 | -182 | 0.72 | 54.2 | 40.37 (15.76) | 18 | 82 | 0.50 | 1.99 (0.74) | 5.06 (2.11) |
| Cyprus | 980 | -24 | -97 | 0.85 | 31.2 | 41.91 (17.43) | 17 | 89 | 0.54 | 2.04 (0.89) | 5.20 (1.85) |
| Ecuador | 1,192 | -100 | -102 | 0.72 | 47.3 | 39.84 (16.13) | 18 | 97 | 0.52 | 2.04 (0.79) | 5.00 (1.88) |
| Estonia | 1,485 | 39 | 108 | 0.85 | 30.8 | 48.47 (18.46) | 18 | 92 | 0.55 | 2.53 (0.83) | 4.36 (1.81) |
| Georgia | 1,158 | -134 | -103 | 0.75 | 34.5 | 44.64 (17.68) | 18 | 85 | 0.54 | 2.64 (0.94) | 3.67 (1.77) |
| Germany | 1,935 | 102 | 46 | 0.92 | 31.7 | 49.59 (17.59) | 17 | 95 | 0.50 | 2.13 (0.88) | 4.82 (1.79) |
| Ghana | 1,552 | -156 | -205 | 0.56 | 43.5 | 30.92 (12.70) | 18 | 82 | 0.50 | 1.61 (0.75) | 4.85 (2.06) |
| Haiti | 1,610 | -39 | -285 | 0.46 | 41.1 | 33.60 (14.83) | 18 | 102 | 0.51 | 2.21 (0.81) | 2.64 (1.48) |
| Hong Kong SAR | 971 | -5 | 199 | 0.91 | 40.1^2^ | 44.56 (16.45) | 18 | 85 | 0.55 | 2.33 (0.79) | 4.72 (1.91) |
| India | 3,981 | -101 | 17 | 0.55 | 35.7 | 41.28 (14.53) | 18 | 92 | 0.44 | 2.09 (0.87) | 4.50 (2.16) |
| Iraq | 1,174 | -99 | -160 | 0.59 | 29.5 | 36.65 (13.39) | 18 | 83 | 0.47 | 2.25 (0.81) | 5.35 (1.86) |
| Japan | 1,786 | 42 | 234 | 0.91 | 32.9 | 51.48 (15.26) | 18 | 80 | 0.48 | 2.44 (0.83) | 4.05 (2.75) |
| Kazakhstan | 1,500 | -106 | 62 | 0.75 | 27.8 | 40.02 (15.35) | 18 | 88 | 0.60 | 2.31 (0.77) | 5.30 (1.95) |
| Jordan | 1,190 | -115 | -120 | 0.70 | 33.7 | 39.82 (15.46) | 18 | 84 | 0.50 | 1.86 (0.84) | 4.99 (2.07) |
| South Korea | 1,180 | 25 | 174 | 0.91 | 31.4 | 43.14 (14.99) | 19 | 85 | 0.51 | 2.06 (0.60) | 5.01 (1.81) |
| Kuwait | 1,070 | -86 | -71 | 0.79 | 35.9^2^ | 36.40 (11.72) | 17 | 79 | 0.34 | 1.73 (0.76) | 5.93 (2.06) |
| Kyrgyzstan | 1,471 | -148 | -49 | 0.62 | 29.0 | 38.78 (14.38) | 18 | 89 | 0.51 | 2.10 (0.71) | 5.56 (2.05) |
| Lebanon | 1,186 | -92 | 1 | 0.75 | 31.8 | 38.48 (14.87) | 18 | 82 | 0.51 | 2.02 (0.77) | 5.83 (1.95) |
| Libya^2^ | 1,977 | -128 | -111 | 0.77 | 32.5 | 38.25 (13.33) | 18 | 78 | 0.48 | 1.72 (0.84) | 5.39 (2.20) |
| Malaysia | 1,299 | -89 | -1 | 0.77 | 41.1 | 40.01 (13.96) | 18 | 80 | 0.49 | 1.76 (0.71) | 6.00 (1.84) |
| Mexico | 1,917 | -63 | -104 | 0.78 | 45.4 | 37.35 (15.02) | 18 | 93 | 0.50 | 2.03 (0.77) | 3.32 (2.44) |
| Morocco | 986 | -103 | -99 | 0.59 | 39.5 | 36.93 (13.17) | 18 | 85 | 0.49 | 1.97 (0.91) | 3.93 (1.86) |
| Netherlands | 1,541 | 182 | 87 | 0.92 | 29.2 | 54.78 (15.82) | 18 | 90 | 0.51 | 2.13 (0.69) | 4.60 (2.29) |
| Nigeria | 1,759 | -291 | -173 | 0.47 | 35.1 | 31.22 (11.69) | 18 | 98 | 0.50 | 1.54 (0.71) | 5.18 (2.13) |
| Pakistan | 1,194 | -171 | -122 | 0.52 | 29.6 | 34.38 (11.86) | 18 | 85 | 0.48 | 1.91 (0.85) | 5.51 (2.13) |
| Peru | 1,145 | -117 | -187 | 0.74 | 43.8 | 39.16 (16.22) | 18 | 88 | 0.49 | 2.35 (0.75) | 4.70 (1.82) |
| Philippines | 1,199 | -126 | -4 | 0.65 | 42.3 | 42.69 (15.55) | 18 | 87 | 0.50 | 2.31 (0.83) | 4.19 (2.47) |
| Poland | 903 | -15 | 9 | 0.82 | 30.2 | 47.88 (17.51) | 19 | 87 | 0.54 | 2.26 (0.90) | 4.48 (1.92) |
| Qatar | 1,035 | -125 | -145 | 0.83 | 40.1^2^ | 37.87 (12.84) | 18 | 93 | 0.54 | 1.62 (0.71) | 6.52 (1.91) |
| Romania | 1,447 | -19 | -64 | 0.79 | 34.8 | 48.28 (17.17) | 18 | 85 | 0.57 | 2.34 (0.84) | 4.78 (2.16) |
| Russia | 2,280 | -21 | 48 | 0.79 | 36.0 | 45.82 (17.35) | 18 | 91 | 0.56 | 2.63 (0.74) | 4.24 (1.76) |
| Rwanda | 1,527 | -134 | -133 | 0.43 | 43.7 | 33.77 (11.23) | 18 | 85 | 0.50 | 1.87 (0.79) | 5.35 (1.83) |
| Singapore | 1,934 | -29 | 89 | 0.90 | 38.3^2^ | 41.89 (16.60) | 18 | 89 | 0.55 | 1.90 (0.71) | 5.70 (1.52) |
| Slovenia | 1,012 | 110 | 15 | 0.89 | 24.4 | 49.15 (17.52) | 18 | 91 | 0.58 | 2.25 (0.93) | 4.92 (1.77) |
| South Africa | 3,404 | -105 | -126 | 0.63 | 63.0 | 36.53 (14.01) | 16 | 85 | 0.50 | 1.74 (0.79) | 5.29 (2.22) |
| Zimbabwe | 1,500 | -143 | -209 | 0.40 | 50.3 | 33.77 (13.51) | 18 | 92 | 0.54 | 1.69 (0.82) | 4.86 (1.92) |
| Spain | 1,017 | 58 | 2 | 0.89 | 34.3 | 46.26 (17.73) | 18 | 99 | 0.51 | 2.10 (0.77) | 4.49 (1.63) |
| Sweden | 1,143 | 133 | 21 | 0.92 | 29.3 | 47.57 (19.17) | 18 | 85 | 0.53 | 1.93 (0.80) | 5.42 (1.85) |
| Thailand | 1,152 | -121 | 87 | 0.69 | 35.0 | 45.28 (12.30) | 18 | 85 | 0.48 | 1.94 (0.76) | 4.57 (2.42) |
| Trinidad and Tobago | 968 | -98 | -134 | 0.76 | 43.2^2^ | 45.72 (17.66) | 18 | 94 | 0.54 | 1.87 (0.89) | 5.00 (2.02) |
| Tunisia | 1,129 | -90 | -102 | 0.71 | 32.8 | 38.36 (15.95) | 18 | 87 | 0.46 | 2.06 (0.87) | 4.68 (2.05) |
| Turkey | 1,524 | -18 | -52 | 0.72 | 41.9 | 38.34 (14.50) | 18 | 86 | 0.51 | 2.15 (0.80) | 5.67 (1.91) |
| Ukraine | 1,488 | 14 | 19 | 0.74 | 25.6 | 47.23 (18.28) | 18 | 89 | 0.60 | 2.75 (0.81) | 4.31 (1.88) |
| Egypt | 1,523 | -141 | -111 | 0.66 | 31.5 | 40.62 (15.26) | 18 | 99 | 0.68 | 2.43 (0.95) | 4.27 (2.01) |
| United States | 2,150 | 33 | 11 | 0.94 | 41.5 | 49.15 (16.79) | 18 | 93 | 0.52 | 1.93 (0.75) | 5.16 (1.91) |
| Uruguay | 933 | 34 | -85 | 0.79 | 40.2 | 44.99 (18.07) | 18 | 88 | 0.53 | 1.96 (0.74) | 4.50 (1.80) |
| Uzbekistan | 1,400 | -113 | -2 | 0.65 | 34.2^2^ | 39.36 (14.68) | 18 | 89 | 0.61 | 2.05 (0.80) | 5.82 (1.76) |
| Yemen | 904 | -99 | -130 | 0.46 | 36.7 | 35.27 (12.88) | 18 | 90 | 0.50 | 2.08 (0.83) | 3.62 (2.15) |

*Notes*. N = sample size; IDV = individualism; FLX = flexibility; HDI = Human Development Index; Gini = Income Gini coefficient; SD = standard deviation. For the individual-level data, numbers outside the parentheses are the means, and numbers inside the parentheses are the standard deviations within each society.

^1^The percentage of female participants; ^2^The Gini coefficients were adopted from the Standardized World Income Inequality Database.
